# Esculetin rebalances M1/M2 macrophage polarization to treat sepsis‐induced acute lung injury through regulating metabolic reprogramming

**DOI:** 10.1111/jcmm.70178

**Published:** 2024-11-13

**Authors:** Feng Chen, Ning Wang, Jiabao Liao, Mengxue Jin, Fei Qu, Chengxin Wang, Min Lin, Huantian Cui, Weibo Wen, Fengjuan Chen

**Affiliations:** ^1^ Department of Critical Care Medicine Jiaxing Hospital of Traditional Chinese Medicine Jiaxing Zhejiang China; ^2^ Yunnan University of Chinese Medicine Kunming Yunnan China; ^3^ Kunming Municipal Hospital of Traditional Chinese Medicine Kunming Yunnan China

**Keywords:** esculetin, fatty acid β‐oxidation, glycolysis, macrophage polarization, metabolic reprogramming, sepsis‐induced acute lung injury

## Abstract

Sepsis‐induced acute lung injury (SALI) is characterized by a high incidence and mortality rate, which has caused a serious medical burden. The pharmacological effects of esculetin (ELT), such as antibacterial and anti‐inflammatory actions, have been widely confirmed. However, the therapeutic effects and mechanisms of ELT on SALI still need to be further clarified. In this study, we first evaluated the therapeutic potential of ELT on a caecal ligation and puncture (CLP) induced septic rat model, particularly in the treatment of acute lung injury. Afterwards, we explored the effect of ELT on macrophage polarization in vivo and in vitro. Then, we investigated the anti‐inflammatory mechanism of ELT based on modulating the metabolic reprogramming of macrophage (the effect on glycolysis in M1, and the effect on fatty acid β‐oxidation in M2). In addition, macrophage metabolic inhibitors (glycolysis inhibitor: 2‐DG, and fatty acid β‐oxidation inhibitor: etomoxir) were used to verify the regulatory effect of ELT on macrophage metabolic reprogramming. Our results proved that ELT intervention could effectively improve the survival rate of SALI rats and ameliorate pathological injury. Next, we found that ELT intervention inhibited M1 polarization and promoted M2 polarization of macrophages in vivo and in vitro, including the downregulation of M1‐related markers (CD86, iNOS), the decrease of pro‐inflammatory factors (nitric oxide, IL‐1β, IL‐6, and TNF‐α), the upregulation of M2‐related markers (CD206, ARG‐1), the increase of immunomodulatory factors (IL‐4 and IL‐10). Subsequently, seahorse analysis showed that ELT intervention inhibited the glycolytic capacity in M1, and promoted the ability of fatty acid β‐oxidation in M2. Besides, ELT intervention inhibited the level of glycolysis product (lactic acid), and the expression of glycolysis‐related genes (*Glut1*, *Hk2*, *Pfkfb1*, *Pkm* and *Ldha*) and promoted the expression of fatty acid β‐oxidation related genes (*Cpt1a*, *Cpt2*, *Acox1*). In addition, we found that the inhibitory effect of ELT on M1 polarization was comparable to that of 2‐DG, while intervention with etomoxir abolished the promoting effect of ELT on M2 polarization. ELT inhibited the inflammatory response in SALI by correcting macrophage polarization (inhibiting M1 and promoting M2). The mechanism of ELT on macrophage polarization was associated with regulating metabolic reprogramming (inhibiting glycolysis in M1 and promoting fatty acid β‐oxidation in M2).

## 1 INTRODUCTION

Sepsis is defined as a life‐threatening organ dysfunction syndrome caused by a dysregulated host response to infection.^1^ Acute lung injury (ALI) is an acute pulmonary interstitial disease and pulmonary edema caused by various pathogenic factors, usually associated with the development of systemic inflammatory reactions. ALI is the most common and fatal complication of sepsis.^2^ A national cross‐sectional survey in China have indicated that the mortality rate of sepsis‐induced ALI (SALI) is close to 40%, reaching approximately 50% in intensive care units.^3^ The high rates of incidence and mortality make SALI a significant medical burden, and lack of effective treatments has heightened the importance of discovering reliable and effective novel therapeutic targets.

Macrophages are a crucial component of the innate immune response. These cells exhibit high plasticity and can polarize into different phenotypes based on the stimuli received. The primary phenotypes are the pro‐inflammatory M1 type and anti‐inflammatory M2 type; they have significant roles in the development of sepsis.^4^, ^5^ Metabolic reprogramming of macrophages is crucial in regulating their pro‐inflammatory/anti‐inflammatory phenotypes.^6^ Previous research has demonstrated metabolic reprogramming in lung tissue during the occurrence of SALI. Glycolysis is significantly activated, leading to the activation of M1 macrophages that secrete numerous pro‐inflammatory factors, promoting the development of SALI. Inhibition of glycolysis can alleviate lung injury caused by SALI.^7^, ^8^ Therefore, modulating metabolic reprogramming to regulate macrophage polarization might serve as a therapeutic target for treating SALI. Inducing fatty acid β‐oxidation could promote the polarization of macrophages toward the M2 phenotype, leading to the secretion of immune regulatory factors and alleviation of SALI symptoms in rats.^9^

In recent years, an increasing number of studies have discovered that natural products from traditional Chinese medicine can regulate macrophage phenotypes by modulating metabolic reprogramming from pro‐ to anti‐inflammatory type. Dehydrocostus lactone (DHL) extracted from *Saussurea involucrata* and *Artemisia argyi* exhibits potent anti‐inflammatory effects. DHL can induce macrophages to polarize from the M1 to M2 phenotype and mitigate lung injury in mice induced by methicillin‐resistant *Staphylococcus aureus* (MRSA).^10^ Xylopic acid glucoside (XPS) possesses strong anti‐inflammatory activity, downregulates the release of pro‐inflammatory factors induced by lipopolysaccharide (LPS), upregulates the secretion of interleukin (IL)‐10, and promotes the polarization of mouse macrophages from the M1 to M2 type.^11^ Curcumin can promote the differentiation of immature T cells to T regulatory cells (Tregs), reducing ALI inflammation and promoting the polarization of macrophages from the M1 to M2 type, possibly due to the production of IL‐10 from Tregs.^12^ Epigallocatechin gallate (EGCG), is a major component of green tea that has anti‐inflammatory and antioxidant effects. EGCG can regulate macrophage polarization toward the M2 phenotype, reduce the expressions of inflammatory factors tumour necrosis factor‐alpha (TNF‐α), IL‐1β, and IL‐6 induced by LPS, and protect mouse lung tissue.^13^ These findings suggest the potential value of natural products from traditional Chinese medicine as therapeutic agents to improve SALI.

Esculetin (ELT) has various pharmacological effects that include anti‐tumour, antioxidant, antimicrobial, anti‐inflammatory and anti‐Alzheimer's activities;^14^ of these all properties, the anti‐inflammatory property is particularly notable. ELT can inhibit the production of inflammatory mediators, such as IL‐6 and TNF‐α, exerting anti‐inflammatory and protective effects in sepsis.^14^ In this study, we hypothesized that ELT can correct macrophage polarization imbalance through metabolic reprogramming and based on this, treat SALI. We explored this hypothesis by establishing a sepsis rat model through cecal ligation and puncture (CLP) to investigate the effects of ELT on SALI improvement and macrophage polarization. Additionally, in vitro experiments were performed to validate ELT's regulatory effects on macrophage polarization and metabolic reprogramming. Finally, macrophage metabolic inhibitors were used to verify ELT's role in regulating macrophage metabolic reprogramming.

## 2 MATERIALS AND METHODS

### 2.1 In vivo experiments

#### 2.1.1 Animals and materials

Male Sprague–Dawley (SD) rats weighing 200 ± 20 g obtained from Beijing Huafukang Biotechnology Co., Ltd. (Production Licence No.: SYXK (Beijing) 2019–0030) were acclimated for 1 week in a quiet environment with a relative humidity of 55%–60%. Rats had access to clean water and food ad libitum. The experiment was approved by the Ethics Committee of Yunnan University of Chinese Medicine (Approval No.: R‐062023G234). Details of the materials and reagents used in this experiment are provided in the Supplementary Materials—Data S1.

#### 2.1.2 Model establishment, grouping and treatment

CLP was used to establish the rat sepsis model.^15^ Under general anaesthesia, the cecum of the rat was exposed through a laparotomy. A ligation of approximately 50% was made, followed by puncture using an 8‐gauge needle, with surgical details following those provided in the literature.^16^

A total of 150 rats were randomly divided into the Sham group, caecal ligation and puncture (CLP) group, low‐dose ELT (L‐ELT) group, medium‐dose ELT (M‐ELT) group, and high‐dose (H‐ELT) group (*n* = 30 per group). The CLP, L‐ELT, M‐ELT and H‐ELT groups underwent CLP to establish the rat model of sepsis. Following the successful establishment of the CLP model, the CLP group will receive intraperitoneal injections of 1 mL of normal saline at 8:00 AM and 8:00 PM daily for seven consecutive days. At the same time points, the L‐ELT, M‐ELT, and H‐ELT groups will receive ELT injections at doses of 25, 50, and 100 mg/kg, respectively, also for 7 days. The ELT dose was set according to previous study.^17^

Subsequently, 15 rats from each group were selected for survival rate analysis. After CLP, the number of surviving rats in each group was recorded daily for seven consecutive days. The remaining 15 rats in each group were euthanized 24 h after modelling, and their lung tissues were collected for efficacy evaluation.

#### 2.1.3 Tissue collection and analysis

After euthanasia, the right lung of each rat was lavaged according to the method described in the literature to collect bronchoalveolar lavage fluid (BALF).^18^ BALF was centrifuged at 4°C for 10 min at 3000 rpm to separate cell pellets and supernatant. Total cell counts in the BALF pellets were determined, and the pellets were used for flow cytometry and macrophage‐related gene expression analysis. The total protein concentration and levels of inflammatory factors in the BALF supernatant were measured.

The left lung of non‐lavaged rats was also collected and divided into two parts. The left lower lobe of the rat's lung was isolated, and the lung slice was immediately weighed (W) after removal. Subsequently, it was dried in an oven at 60°C for 48 h and reweighed (D) to calculate the wet‐to‐dry weight ratio of the rat's lung tissue (W/D).^19^ The upper part was fixed with formalin and used for subsequent pathological and immunofluorescence analyses.

#### 2.1.4 Histological staining

The upper part of the left lung was embedded in paraffin and sectioned at a thickness of 3 μm. The sections were routinely stained by haematoxylin and eosin (HE). Pathological changes in lung tissues of each group were observed by optical microscopy. This study utilized the scoring criteria for the degree of alveolitis as reported by Szapiel et al.,^20^ with analysis conducted under a 400x magnification. 0 points: No alveolitis; 1 point: Mild alveolitis, with the affected area constituting less than 20% of the entire lung and normal alveolar structure; 2 points: Moderate alveolitis, with the affected area ranging from 20% to 50%, with more severe involvement near the pleura; 3 points: Severe alveolitis, with the affected area exceeding 50%, and occasional presence of mononuclear cells within the alveolar spaces.

#### 2.1.5 Immunofluorescence staining

After dewaxing the sections from the previous step, they were gradually hydrated using decreasing concentrations of ethanol. Following antigen retrieval, the sections were incubated with a 3% hydrogen peroxide solution at 25°C to block endogenous peroxidase activity. Next, the sections were blocked with goat serum for 1 h, then incubated with a primary antibody against the target protein at 4°C for 12 h. This was followed by an incubation with the appropriate secondary antibodies at 25°C for 2 h. The nuclei of the cells were stained with 4′,6‐diamidino‐2‐phenylindole (DAPI). The area of positive expression was quantified using Image Pro Plus 6.0. Immunofluorescence staining was performed using mouse anti‐CD11b (#ab1211, dilution 1:500), purchased from Abcam (Cambridge, MA, USA), and rabbit anti‐CD206 (#18704‐1‐AP, dilution 1:500) and rabbit anti‐CD86 (#13395‐1‐AP, dilution 1:500), both purchased from Proteintech Group, Inc. (Chicago, USA).

### 2.2 In vitro experiments

#### 2.2.1 Cells

Bone marrow‐derived macrophages (BMDMs) were isolated and induced from the femur and tibia bone marrow of male rats (12‐weeks‐of‐age, 300–350 g) following a previously established protocol for rat BMDM in vitro isolation and culture.^21^ Briefly, after euthanasia, the intact femur and tibia were quickly removed. The dry end of the bone was trimmed and the bone marrow cavity was alternately washed with DMEM from both ends. The flushing liquid was collected, centrifuged at 800 rpm for 8 min, and the supernatant was discarded. After resuspension and centrifugation following cell sieving, the material was resuspended in an appropriate amount of red blood cell lysis buffer. The suspension was left for 5 min, centrifuged at 800 rpm for 8 min, resuspended, washed three times with PBS, and resuspended in DMEM containing 10% heat‐inactivated fetal calf serum, and 100 ng/mL macrophage‐colony stimulating factor. Cells were cultured in a conventional incubator. Cell growth was observed by inverted phase contrast microscopy and documented. When cell fusion reached 80%–90%, cells were digested using 0.25% trypsin and 0.1% EDTA for subsequent experiments.

To investigate the effect of the drug on BMDM polarization, the obtained BMDMs were seeded into 24‐well plates at a density of 2 × 10^5^ cells per well. The cells were then incubated in M‐CSF‐free medium for 24 h, followed by washing the cells. Afterward, the cells were stimulated with 100 ng/mL LPS and 20 ng/mL IFN‐γ for 24 h, with or without drug intervention. After incubation, both the cells and supernatants were collected separately.

#### 2.2.2 Cell viability assay

The cytotoxicity of ELT was evaluated using the MTT assay. Seed BMDM cells at a density of 5 × 10^3^ cells per well in a 96‐well plate and incubate for 24 h. The cells were then treated with different concentrations of ELT (0, 10, 25, 50, 100, and 200 μM) for 24 h. After incubation, 10 μL of MTT solution (5 mg/mL) was added to each well. After an additional 4 h of incubation, the culture medium was gently removed and 100 μL of dimethyl sulfoxide was added. The absorbance of the samples was measured at 570 nm.

#### 2.2.3 Cellular metabolism analysis

Cellular metabolism changes were measured using a Seahorse energy metabolism analyser (Agilent, San Diego, CA, USA). BMDMs were seeded at a density of 8 × 10^4^ cells/well in Seahorse XF 24 plates (Agilent). M1/M2 polarizing inducers and high‐dose ELT were added to the cells as described in section 2.2.1, followed by overnight incubation. To measure glycolytic capacity, the extracellular acidification rate (ECAR) was measured in XF Seahorse medium (Agilent) containing 2 mM glutamine. The concentrations of metabolic modulators were glucose (30 mM), oligomycin (2 μM), and 2‐deoxy‐d‐glucose (2‐DG, 100 mM).^22^ To assess exogenous and endogenous fatty acid oxidation (FAO), cells were cultured overnight in DMEM containing 0.5 mM glucose, 1 mM GlutaMAX, 0.5 mM carnitine, and 1% fetal bovine serum, followed by replacement of the medium with FAO assay medium to measure the oxygen consumption rate. The concentrations of metabolic modulators were palmitate‐bovine serum albumin (175 μM), oligomycin (2 μM), carbonyl cyanide‐p‐trifluoromethoxyphenylhydrazone (FCCP, 4 μmol/L), and etomoxir (80 μM).^23^

#### 2.2.4 FAO assay

Cell homogenates were collected 24 h after drug intervention to assess fatty acid β‐oxidation using a commercial assay kit with Fatty acid oxidation assay kit (#BR00001) were purchased from AssayGenie (Ireland).

#### 2.2.5 Detection of lactate and nitric oxide

24 h after drug intervention, cell supernatants were collected, and a commercial assay kit was used to detect lactate and nitric oxide levels.

### 2.3 Enzyme‐linked immunosorbent assay

Enzyme‐linked immunosorbent assay (ELISA) was used to test the levels of IL‐1β, IL‐6, IL‐4, IL‐10, and TNF‐α in both BALF and cell supernatants. Specific operational procedures were conducted following the instructions provided with the respective ELISA kits.

### 2.4 Flow cytometry

The cells were collected and centrifuged at 6000 g for 5 min at room temperature. To measure the expression levels of CD86, CD11b and CD206 in both collected BALF cell pellets and cells, the cells were stained using anti‐rat CD11b (FITC‐65229), rabbit anti‐CD206 (18704‐1‐AP) antibodies, PE‐conjugated anti‐CD86 (12–0860‐83) and APC‐conjugated goat anti‐rabbit IgG (H + L) (A‐10931) at 4°C for 30 min in the dark environment. All samples were acquired on a BD C6 flow cytometer (BD Bioscences) and analysed with FlowJo software.

### 2.5 Quantitative PCR (qPCR)

Total RNA was extracted from BALF cell pellets and cells cultured in vitro using TRIzol® (Life Technologies, Inc., USA), according to the manufacturer's instructions; the RNA (1 μg) from each group was reverse‐transcribed by a reverse transcription kit (ReverTra Ace® qPCR RT Kit, Toyobo Inc., Japan) to obtain the corresponding cDNA. qPCR was performed to determine the mRNA expression levels of the target genes. The relative expression of each target mRNA to β‐actin was calculated using the 2−^ΔΔ^CT method. Primer sequences can be found in the Supplementary Materials—Data S1.

### 2.6 Western blotting

Collected cells were placed in ice‐cold RIPA buffer, and total protein was extracted using ultrasonic homogenization. Protein concentration in the supernatant was determined using the bicinchoninic acid method. After adding protein loading buffer, the samples were heated at 99°C for 5 min to denature the proteins. Denatured proteins were then separated by SDS‐PAGE, followed by transfer to a PVDF membrane. The membrane was blocked with a solution containing 5% skim milk for 1 h at 4°C, followed by overnight incubation at 4°C with primary antibodies against the target proteins: iNOS (1:1000), ARG‐1 (1:5000), rabbit anti‐CD206 (1:1000), and rabbit anti‐CD86 (1:4000). After three washes with TBST, the membrane was incubated with horseradish peroxidase‐conjugated secondary antibody goat anti‐rabbit IgG H&L (abcam, ab205718) at 25°C for 2 h. Following another round of washing, chemiluminescence was induced using ECL reagents, and band quantification analysis was performed using the Image J (version 1.52a, NIH, Bethesda, Maryland, USA).

### 2.7 Statistical analyses

Data were expressed as the mean ± the standard deviation, and statistical analysis was done using SPSS software (Version 22.0). For comparisons between two groups, a two‐tailed unpaired Student's *t*‐test was used, while one‐way analysis of variance (ANOVA), followed by Tukey's multiple comparisons test was used with adjustment for multiplicity among three or more groups. Survival curves were calculated according to the Kaplan–Meier method; survival analysis was performed using the log rank test. Differences with *p* value <0.05 were considered statistically significant.

## 3 RESULTS

### 3.1 ELT alleviates lung injury in CLP‐induced SALI rat model

**FIGURE 1:**
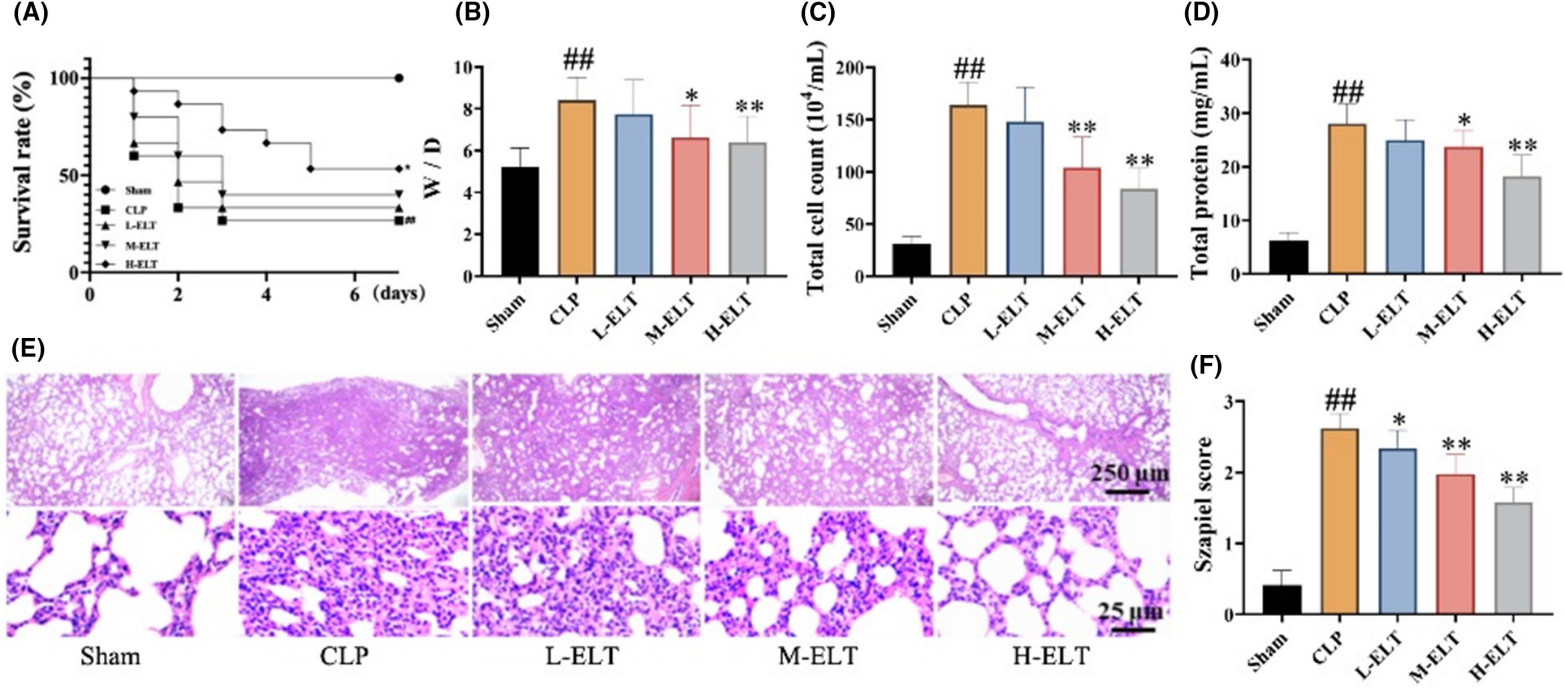
ELT improved lung injury in CLP‐induced SALI rats. The Kaplan–Meier survival curves demonstrate a significantly higher survival rate (A) in the H‐ELT groups of sepsis rats. Additionally, ELT intervention can markedly reduce the lung wet/dry weight ratio (B), total cell count (C), and total protein content (D) in BALF. HE staining showed that ELT intervention could reduce the lung injury score in SALI rats (E, F). The initial number of rats for survival statistics was 15 per group. In subsequent experiments, sham, CLP, L‐, M‐, H‐ELT (*n* = 15, 8, 7, 10, 11 per group) groups. ##*p* < 0.01 versus sham group, *p < 0.05 versus the CLP group, **p < 0.01 versus the CLP group.

The 7‐day survival rate was 100% for the Sham group and 27% for the CLP group. ELT intervention significantly increased the survival rate of sepsis‐induced rats, with survival rates of the L‐ELT, M‐ELT, and H‐ELT groups being 33%, 40%, and 53%, respectively (Figure 1A).

Following modelling and drug administration, the lung wet‐to‐dry weight ratio (W/D), total cell count, and total protein content in the BALF of the CLP group rats were significantly increased. After ELT intervention, these indicators were reduced to varying degrees (Figure 1B–1D). HE staining revealed thickened alveolar walls, unclear alveolar structures, alveolar dilation with inflammatory cell infiltration, and extensive bronchial epithelial cell necrosis in CLP rats. Following ELT intervention, alveolar structures tended to be more intact, and inflammatory cell numbers were significantly reduced, indicating reduced severity of lung injury (Figure 1E,F).

### 3.2 Effects of ELT on macrophage polarization in CLP‐induced SALI rat model

**FIGURE 2:**
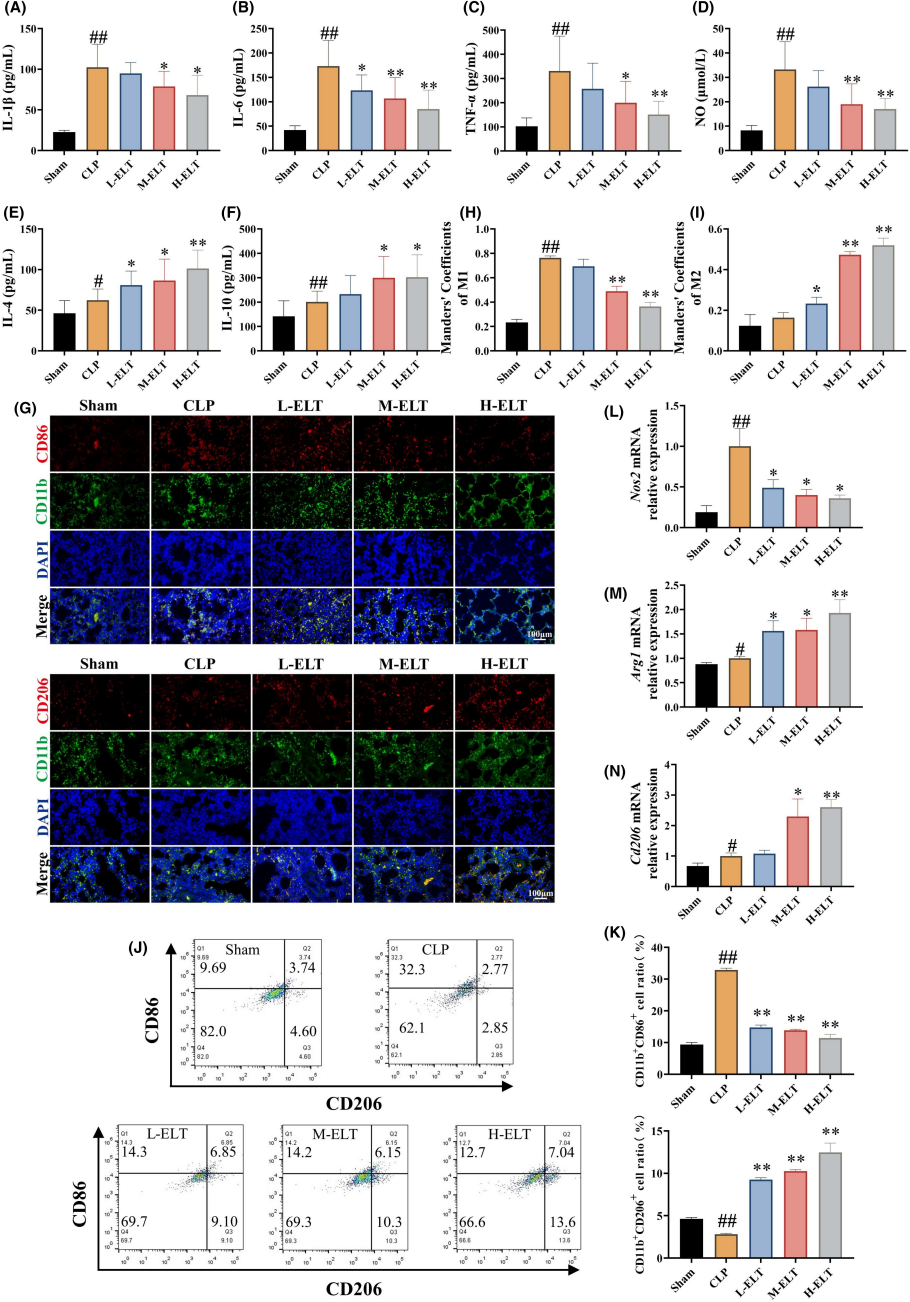
ELT intervention inhibited M1 and upregulated M2 polarization of macrophages in SALI rats. ELT intervention decreased the levels of M1 related factors: IL‐1β (A), IL‐6 (B), TNF‐α (C), nitric oxide (D), and increased the levels of M2 related factors: IL‐4 (E), IL‐10 (F). Immunofluorescence (G–I) and flow cytometry (J, K) results showed that ELT intervention reduced the expression of CD11b^+^CD86^+^ and increased the expression of CD11b^+^CD206^+^. qPCR results showed that mRNA level of *Nos2* (L) was downregulated, and mRNA levels of *Arg1* (M) and *Cd206* (N) were increased after ELT intervention. Data were shown as the mean ± SD with *n* = 15, 8, 7, 10, 11 per group, and the significant markers were consistent with the previous. #*p* <  0.05 versus sham group, ##*p* < 0.01 versus sham group, *p < 0.05 versus the CLP group, **p < 0.01 versus the CLP group.

Examination of M1 and M2‐related factors in BALF revealed a significant increase in M1‐related factors (IL‐1β, IL‐6, TNF‐α, and NO) and M2‐related factors (IL‐4 and IL‐10) in CLP rats (Figure 2A–2F). After ELT intervention, M1‐related factors were significantly reduced and M2‐related factors were significantly increased, with the highest dose of ELT showing the most prominent effects. Immunofluorescence and flow cytometry results indicated elevated expressions by CD11b^+^CD86^+^ cells in lung tissues and BALF of SALI rats, while the levels of CD11b^+^CD206^+^ cells in lungs and BALF were similar as compared to those in Sham group (Figure 2G–2K). Following ELT intervention, the expression level of CD11b^+^CD86^+^ cells was notably reduced, while the expression level of CD11b^+^CD206^+^ was significantly increased. Then, qPCR experiments were performed to further validate the impact of ELT on macrophage polarization. The results demonstrated that ELT downregulated induced NO synthase (iNOS) mRNA expression and upregulated the expression of arginase‐1 (ARG‐1) and CD206 mRNA (Figure 2L–2N). These findings suggested that ELT intervention inhibited M1 polarization of macrophages in SALI rats and simultaneously promoted M2 polarization.

### 3.3 Effects of ELT on rat BMDM polarization in vitro

**FIGURE 3:**
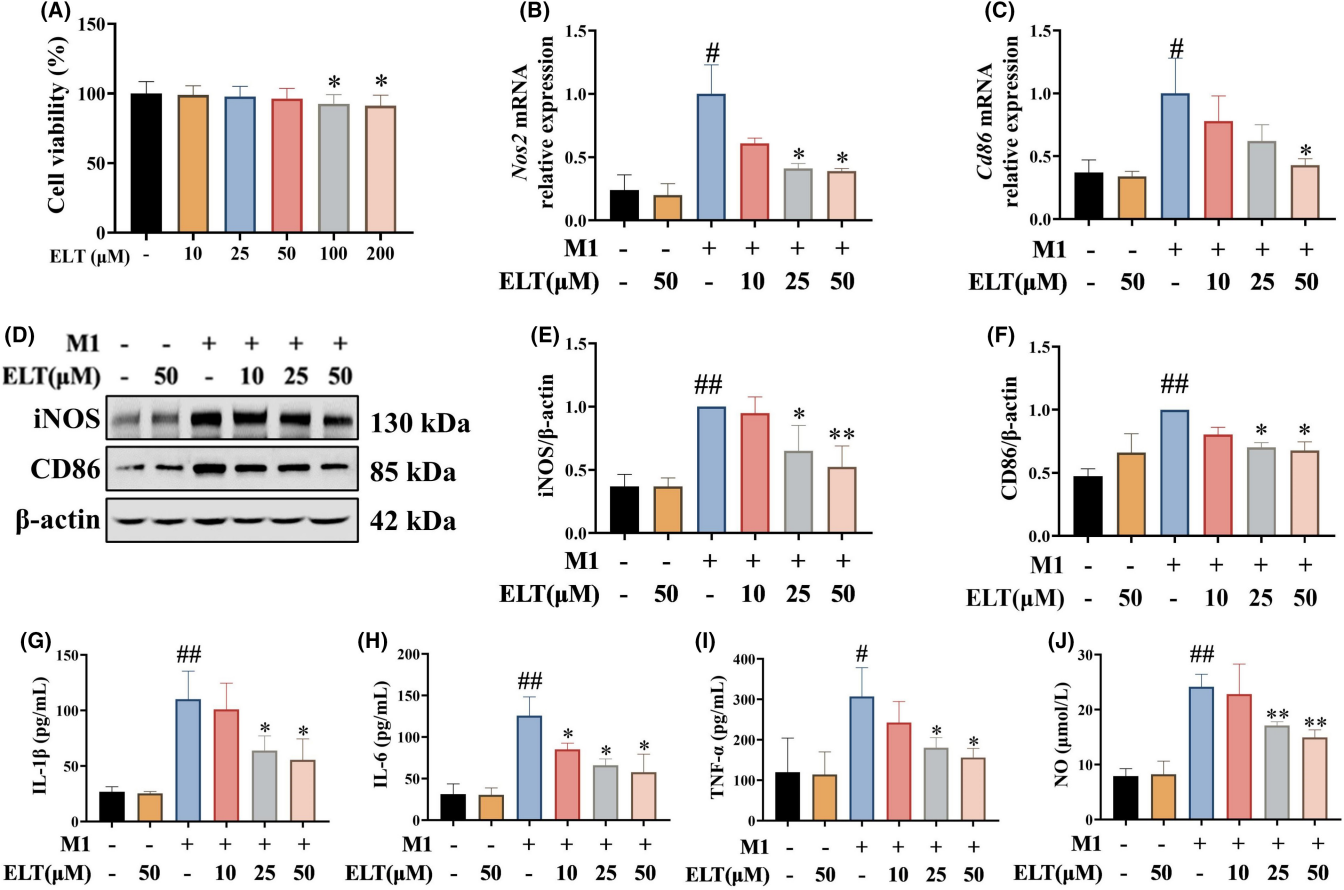
ELT intervention inhibited M1 polarization of BMDM in vitro. The MTT assay (A) showed that ELT with 10, 25, and 50 μM had no effect on BMDM activity. LPS (100 ng/mL) and IFN‐γ (20 ng/mL) were used to induce M1 polarization of BMDM, and ELT intervention with 10, 25, 50 μM was performed. qPCR indicated that mRNA levels of *Nos2* (B) and *Cd86* (C) were decreased, and, western blotting (D) indicated that levels of iNOS (E) and CD86 (F) were downregulated in BMDM after ELT intervention. Furthermore, ELT intervention decreased the levels of IL‐1β (G), IL‐6 (H), TNF‐α (I), and nitric oxide (J). Data were shown as the mean ± SD with *n* = 3. #*p* < 0.05, ##*p* < 0.01 versus M1(−)ELT(−) group, *p < 0.05, **p < 0.01 versus the M1(+)ELT(−) group.

The regulatory mechanism of ELT on macrophage polarization were investigated in vitro. MTT assays of BMDMs following primary isolation and cultivation was employed to assess the cytotoxicity of ELT on rat BMDMs (Figure 3A). The results showed that ELT at concentrations of 10, 25, and 50 μM had no significant impact on rat BMDM viability. These ELT concentrations were chosen for the study of BMDM polarization.

BMDM polarization toward M1 macrophages was induced using a combination of LPS and IFN‐γ, with varying concentrations of ELT. qPCR and western blotting analyses indicated that the expressions of iNOS and CD86 increased in BMDMs induced by LPS and IFN‐γ, confirming the successful induction of M1 macrophages (Figure 3B–3F). Different doses of ELT led to varying degrees of reduction in iNOS and CD86 expressions. Furthermore, the levels of inflammatory factors IL‐1β, IL‐6, TNF‐α, and iNOS in the supernatant of BMDMs induced by LPS and IFN‐γ were significantly elevated compared to those in the M1(−)ELT(−) group (Figure 3G–3J). ELT dose‐dependently reduced the levels of these inflammatory factors compared with M1(+)ELT(−) group. These results revealed the inhibitory effect of ELT on the M1 polarization of macrophages.

**FIGURE 4:**
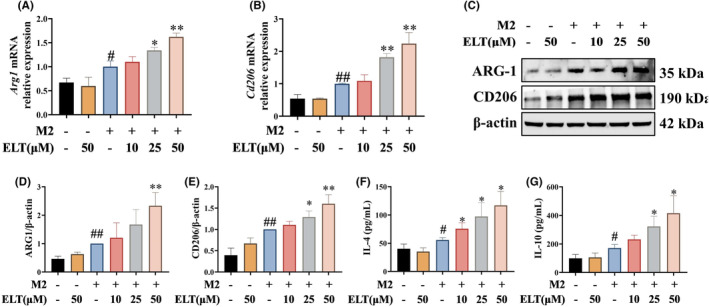
ELT intervention promoted M2 polarization of BMDM in vitro. IL‐4 (20 ng/mL) were used to induce M2 polarization of BMDM, and ELT intervention with 10, 25, 50 μM was performed. qPCR suggested that mRNA levels of *Arg1* (A) and *Cd206* (B) were increased, and, western blotting (C) results revealed levels of ARG‐1 (D) and CD206 (E) were upregulated in BMDM after ELT intervention. Furthermore, ELT intervention increased the levels of IL‐4 (F) and IL‐10 (G). Data were shown as the mean ± SD with *n* = 3. #*p* < 0.05, ##*p* < 0.01 versus M2(−)ELT(−) group, *p < 0.05, **p < 0.01 versus the M2(+)ELT(−) group.

Additionally, BMDM polarization toward M2 macrophages was induced using IL‐4 and different concentrations of ELT. qPCR and western blotting indicated that the expressions of ARG‐1 and CD206 were increased in IL‐4‐induced BMDM cells, indicating the successful induction of M2 macrophages (Figure 4A–4E). ELT dose‐dependently upregulated the expression of ARG‐1 and CD206. Moreover, supernatants of IL‐4‐induced BMDM cultures displayed significantly elevated levels of immune regulatory factors IL‐4 and IL‐10 compared to those in the M2(−)ELT(−) group (Figure 4F,G). ELT consistently increased the levels of these immune regulatory factors in a dose‐dependent pattern compared with M2(+)ELT(−) group. These results suggested that ELT promoted M2 macrophage polarization.

### 3.4 Effects of ELT on rat BMDM metabolic reprogramming

Metabolic reprogramming has been shown with crucial role during macrophage polarization. We hypothesized that ELT might regulate macrophage polarization through metabolic reprogramming. Based on earlier findings that the highest dose of ELT exhibited optimal therapeutic effects, the current high dose was used for the verification experiment.

**FIGURE 5:**
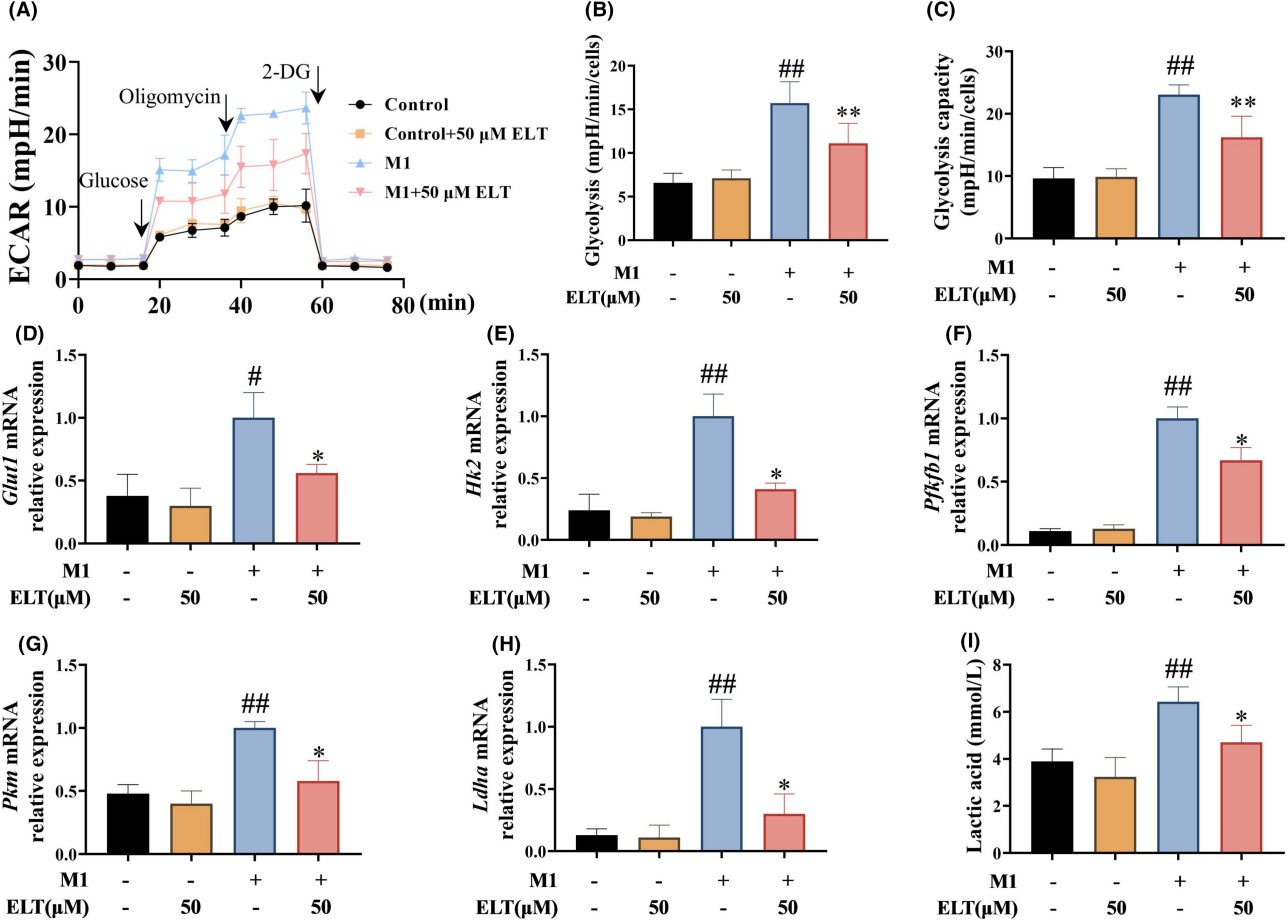
ELT intervention inhibited BMDM glycolysis in vitro. LPS (100 ng/mL) and IFN‐γ (20 ng/mL) were used to induce M1 polarization of BMDM, and ELT intervention with 50 μM was performed. The ECAR (A) of the BMDM was measured by Seahorse XF analysis. For ECAR, the cells were treated by sequential injection of the following compounds: Glucose (30 mM), oligomycin (2 μM), and 2‐deoxyglucose (2‐DG, 100 mM). The glycolysis (B) and glycolytic capacity (C) were evaluated in (A). RT‐qPCR results showed that glycolysis related mRNA levels of *Glut1* (D), *Hk2* (E), *Pfkfb1* (F), *Pkm* (G) and *Ldha* (H) were decreased after ELT intervention. Furthermore, ELT intervention decreased the level of lactic acid (I). Data were shown as the mean ± SD with *n* = 3, and the significant markers are consistent with Figure 3.

**FIGURE 6:**
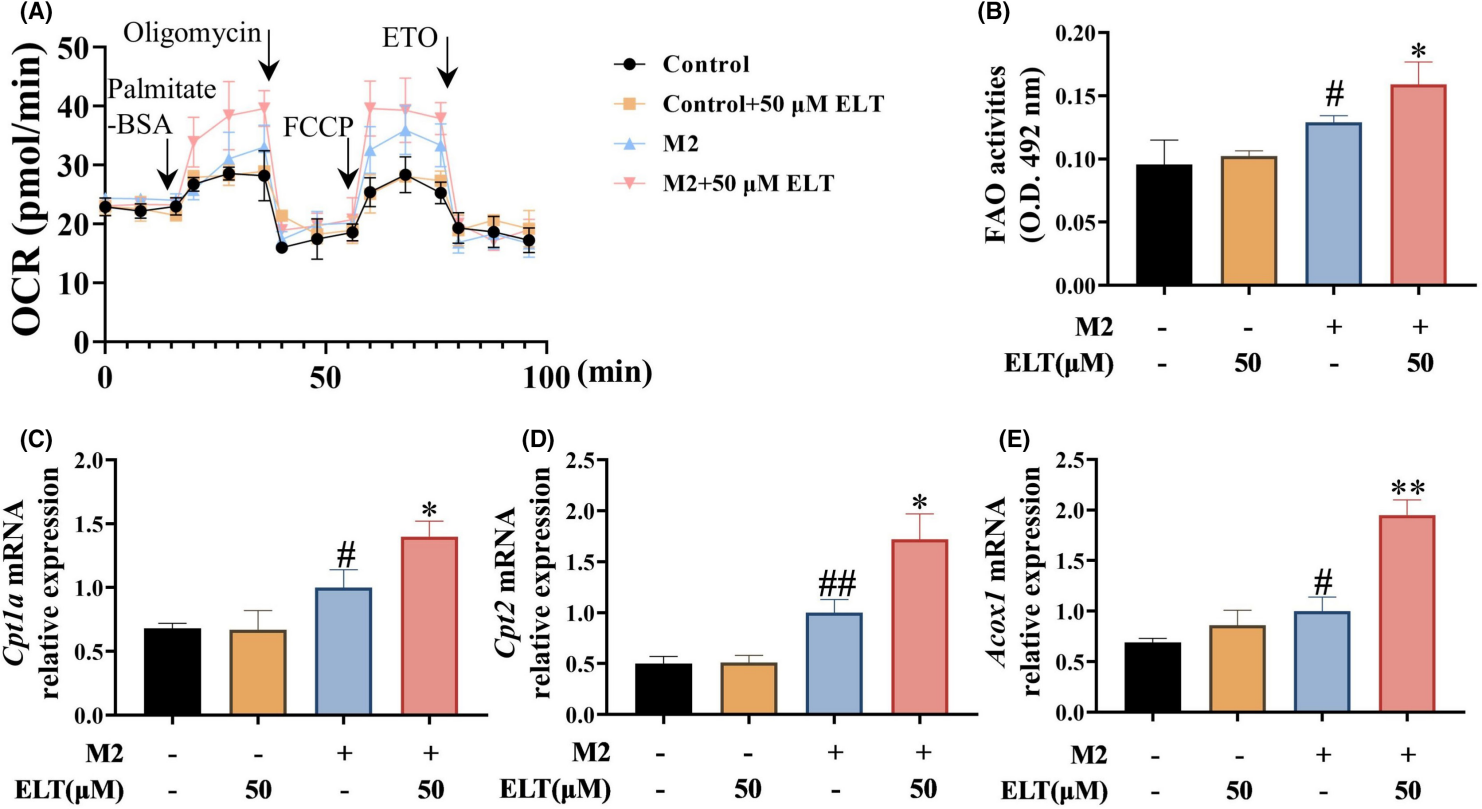
ELT intervention promoted fatty acid β‐oxidation in BMDM in vitro. LPS (100 ng/mL) and IFN‐γ (20 ng/mL) were used to induce M1 polarization of BMDM, and ELT intervention with 50 μM was performed. The OCR (A) of the BMDM was measured by Seahorse XF analysis. For OCR, the cells received sequential injection of the following compounds: Palmitate‐BSA (175 μM), oligomycin (2 μM), carbonyl cyanide‐4 (trifluoromethoxy) phenylhydrazone (FCCP, 4 μM) and etomoxir (ETO, 80 μM). The FAO activity (B) was increased after ELT intervention. RT‐qPCR results showed that fatty acid β‐oxidation related mRNA levels of *Cpt1a* (C), *Cpt2* (D), and *Acox1* (E) were increased after ELT intervention. Data were shown as the mean ± SD with *n* = 3, and the significant markers are consistent with Figure 4.

During the induction of M1 macrophage polarization, LPS combined with IFN‐γ induced significant increases in glycolysis and glycolytic capacity in BMDMs (Figure 5A–5C), along with elevated expressions of the glycolysis‐related genes (*Glut1*, *Hk2*, *Pfkfb1*, *Pkm* and *Ldha*), and an increase in lactate levels in the supernatant (Figure 5D–5I). After high‐dose ELT intervention, these indicators exhibited varying degrees of reduction. When inducing M2 macrophage polarization, IL‐4 increased the capacity for fatty acid β‐oxidation in BMDMs (Figure 6A,B), accompanied by elevated expressions of genes related to fatty acid β‐oxidation (*Cpt1a*, *Cpt2*, *and Acox1*) (Figure 6C–6E). In the presence of the high‐dose of ELT, these indicators were further upregulated to varying degrees.

**FIGURE 7:**
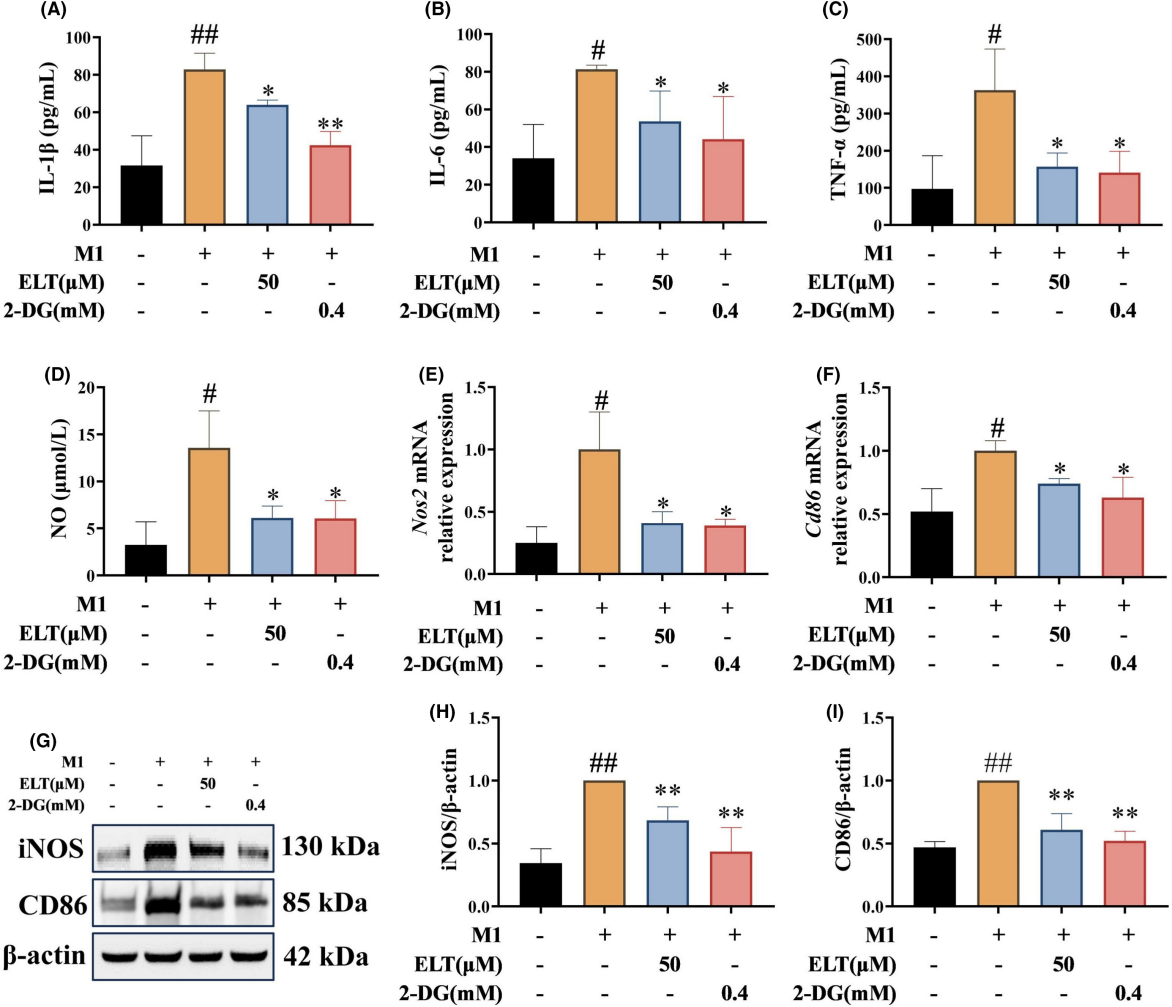
ELT and glycolysis inhibitor 2‐DG had similar efficacy in vitro. LPS (100 ng/mL) and IFN‐γ (20 ng/mL) were used to induce M1 polarization of BMDM, and ELT intervention with 50 μM and 2‐DG intervention with 0.4 mM were performed. ELT and 2‐DG intervention decreased the levels of IL‐1β (A), IL‐6 (B), TNF‐α (C), and nitric oxide (D). Furthermore, qPCR suggested that mRNA levels of Nos2 (E) and Cd86 (F) were decreased, and, western blotting (G) suggested that levels of iNOS (H) and CD86 (I) were downregulated in BMDM after ELT and 2‐DG intervention. Data were shown as the mean ± SD with n = 3, and the significant markers are consistent with Figure 3.

**FIGURE 8:**
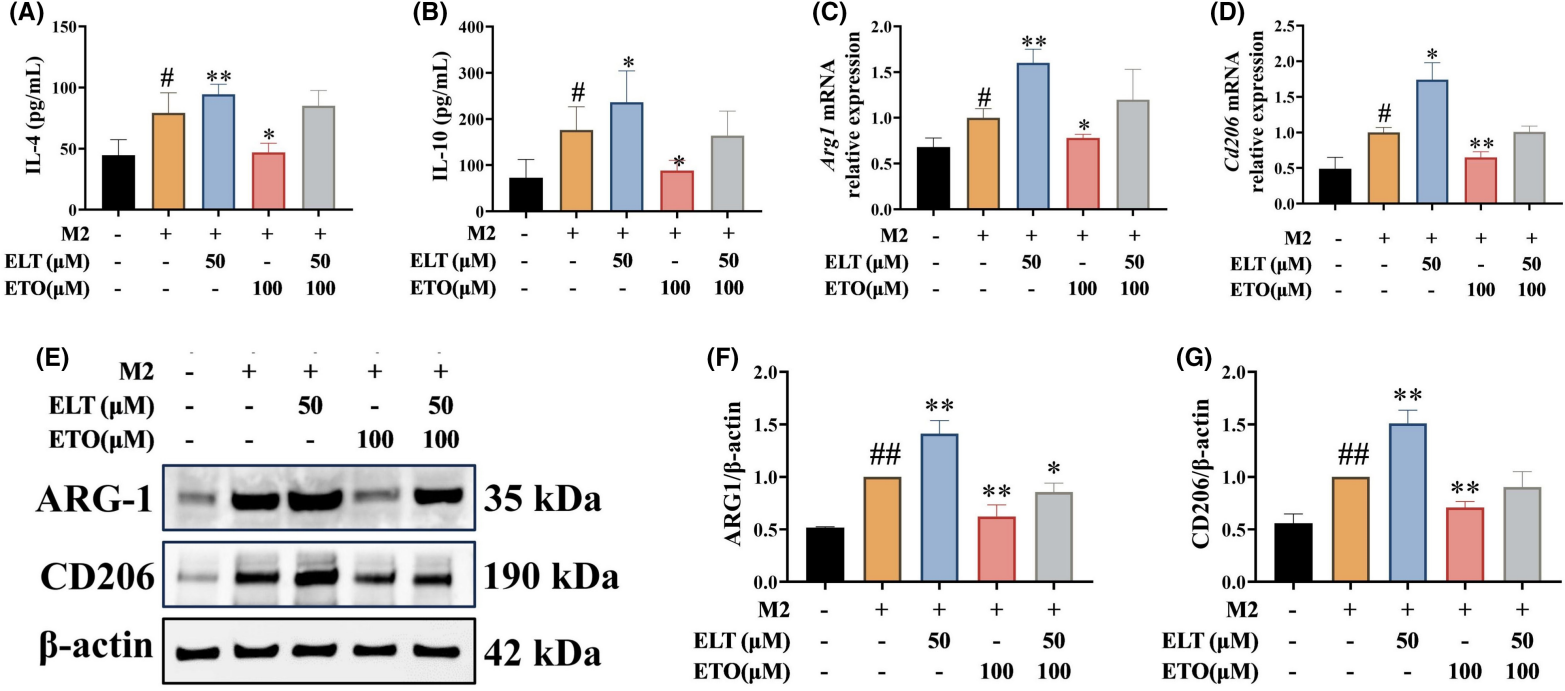
ELT intervention promoted M2 polarization, whereas the ETO intervention abolished it in vitro. IL‐4 (20 ng/mL) were used to induce M2 polarization of BMDM, then ELT, ETO and ELT + ETO intervention with 50, 100, and 50 + 100 μM was performed. (A–G) The levels of IL‐4 (A), IL‐10 (B), *Arg1* (C), *Cd206* (D), ARG‐1 (F) and CD206 (G) were upregulated in BMDM after ELT intervention, whereas were downregulated after ETO intervention based on ELT intervention. Data were shown as the mean ± SD with *n* = 3, and the significant markers are consistent with Figure 4.

To further investigate the impact of ELT on rat BMDM metabolic reprogramming, 2‐DG (a glycolysis inhibitor) was chosen as a positive control for verification. In the presence of the high‐dose of ELT and 2‐DG, levels of IL‐1β, IL‐6, TNF‐α, and NO in the supernatant, and iNOS and CD86 in cells were significantly reduced (Figure 7). High‐dose ELT and 2‐DG exhibited comparable inhibitory effects on M1 polarization. Furthermore, we used etomoxir (ETO, a fatty acid β‐oxidation inhibitor) to evaluate whether ELT induced M2 polarization by promoting FAO. The combination of IL‐4, ELT, and etomoxir did not significantly affect the expressions of ARG‐1 and CD206 in cells, or the levels of IL‐4 and IL‐10 in supernatants (Figure 8), suggesting that etomoxir abolished the promoting effect of ELT on M2 polarization.

## 4 DISCUSSION

ALI is a common complication in sepsis patients and a significant contributor to sepsis‐related mortality.^24^ The underlying mechanisms of SALI have been intensively studied. ELT has been implicated as a potentially valuable therapy for ALI.^17^ However, its specific mechanisms of action remain unclear. This study explored the specific protective effects of ELT on SALI and its potential mechanisms from the perspective of macrophage metabolic reprogramming. The CLP model is the most commonly used animal model for SALI, closely resembling the clinical course and progression of SALI.^25^ After CLP, a cytokine storm occurs, leading to increased intestinal permeability, gut microbiota translocation, and a series of inflammatory reactions, exacerbating intestinal barrier dysfunction and ultimately triggering and promoting the development of SALI.^26^ In our study, lung tissue from SALI rats exhibited characteristics, such as significant inflammatory cell infiltration, evident disruption of alveolar structure, and thickened alveolar walls. At the same time, lung edema and permeability increased, indicating the successful establishment of the septic rat model. Following ELT intervention, these symptoms were markedly improved, with the highest dosage group showing the best efficacy. However, the underlying mechanism remained unclear and warranted further investigation.

Macrophages are primary innate immune effector cells in the lungs. They can modify their functions and phenotypes based on environmental signals and play a crucial role in initiating and resolving lung inflammation.^27^ Therefore, we measured the regulatory effect of ELT on macrophage polarization. Macrophages can be broadly divided into two subtypes according to their characteristics and functions: classically activated macrophages (M1) and alternatively activated macrophages (M2).^28^ M1 macrophages express iNOS and CD86, are activated by external stimuli, and secrete a plethora of pro‐inflammatory cytokines, including IL‐1β, IL‐6, TNF‐α, and nitric oxide.^29^ Conversely, M2 macrophages express CD206 and ARG1, secrete immunoregulatory factors, such as IL‐4 and IL‐10, suppress tissue and cellular inflammation, and facilitate tissue repair and vascular formation.^30^ M1 and M2 macrophages maintain a dynamic equilibrium in the body to stabilize the internal environment. However, in the early stages of sepsis, M1 macrophages become excessively activated, while M2 macrophages are only minimally activated, causing a rapid increase in the ratio of pro‐inflammatory mediators, thereby inducing the development of ALI. The in vivo and in vitro experiments in the present study revealed the ELT‐mediated downregulated expressions of inflammatory factors IL‐1β, IL‐6, TNF‐α, and nitric oxide, as well as M1 markers (iNOS and CD86), and upregulated expressions of immunoregulatory factors IL‐4, IL‐10, as well as M2 markers (ARG‐1 and CD206), effectively alleviating SALI.

Metabolic reprogramming has been demonstrated with important regulatory role in the process of macrophage polarization.^31^ Thus, we then investigated the influence of ELT on macrophage metabolism. Immune‐activated M1 macrophages typically sustain efficient glycolysis for energy supply,^32^ the promotion of glycolysis induces M1 macrophage polarization.^33^ Glucose uptake in glycolysis is primarily facilitated by the glucose transporter 1 (GLUT1) uniporter.^34^ Overexpression of GLUT1 in LPS‐induced M1 murine macrophages can enhance glycolytic activity and pro‐inflammatory cytokine production.^35^ Glucose phosphorylation to glucose‐6‐phosphate is catalysed by hexokinase (HK) to initiate glycolysis.^36^ Moreover, HK can be competitively bound by degradation products of bacterial cell walls, triggering inflammatory responses.^36^, ^37^ Phosphofructokinase 1 (PFK1) plays a bridging role during the glycolytic pathway. It catalyses the conversion from fructose‐6‐phosphate to fructose‐2,6‐bisphosphate, which enhances glycolytic flux.^31^ The final rate‐limiting step of glycolysis is the conversion of phosphoenolpyruvate to pyruvate, catalysed by pyruvate kinase (PK). PK subtype PKM2‐mediated glycolysis may promote inflammasome activation.^38^ In addition, lactate denhydrogense A (LDHA) expression is upregulated in M1 macrophages, promoting the conversion of pyruvate to lactate.^31^, ^39^ Cellular metabolism analyses in the present study demonstrated increased levels of glycolysis in macrophages induced for M1 polarization. ELT reduced the levels of glycolytic indicators, suggesting the inhibitory effect of ELT on M1 macrophage glycolysis. M2 macrophages often display enhanced fatty acid β‐oxidation capability, generating abundant ATP as a vital energy source. By inducing fatty acid β‐oxidation, M2 macrophage polarization can be promoted.^40^ Carnitine palmitoyl transferase (CPT) is key in the fatty acid β‐oxidation process. Upon the transport of long‐chain fatty acids into the cell, fatty acids are activated to acyl‐CoA at the cytosolic site. CPT1 converts the acyl‐CoA into acylcarnitine, facilitating transport through the mitochondrial membrane. Subsequently, CPT2 converts the acylcarnitine back into acyl‐CoA, which participates in the following fatty acid β‐oxidation process. The absence of both CPT1 and CPT2 impedes progression of fatty acid β‐oxidation.^41^ Acyl‐CoA oxidase 1 (ACOX1), the enzyme catalysing the first step of fatty acid β‐oxidation, also serves as a rate‐limiting enzyme in this process, accelerating fatty acid β‐oxidation when highly expressed.^42^ Similarly, during the induction of M2 polarization in the present study, an elevation in fatty acid β‐oxidation levels within macrophages was observed throughout the experiment. Moreover, the enzymes associated with fatty acid β‐oxidation were upregulated. ELT intervention further upregulated these indicators, indicating its promotion of M2 macrophage fatty acid β‐oxidation.

To further verify whether ELT regulates macrophage polarization through metabolic reprogramming, 2‐DG (a glycolysis inhibitor) was employed as a positive control during M1 macrophage polarization. A prior study reported that after 24 h of 0.4 mM 2‐DG incubation, the expression of inflammatory factors and M1 markers were decreased in LPS‐induced M1 macrophages.^39^ Our results showed that high‐dose ELT and 2‐DG exhibited similar effects on M1 polarization. Additionally, we employed etomoxir (a fatty acid β‐oxidation inhibitor) during M2 polarization to validate the impact of ELT on M2 polarization. In a previous study, 100 μM of etomoxir inhibited M2 macrophage polarization by blocking fatty acid β‐oxidation.^43^ Similarly, our results suggest that the promotion of M2 macrophage polarization by ELT was weakened or eliminated by inhibiting fatty acid β‐oxidation.

## 5 CONCLUSION

**FIGURE 9:**
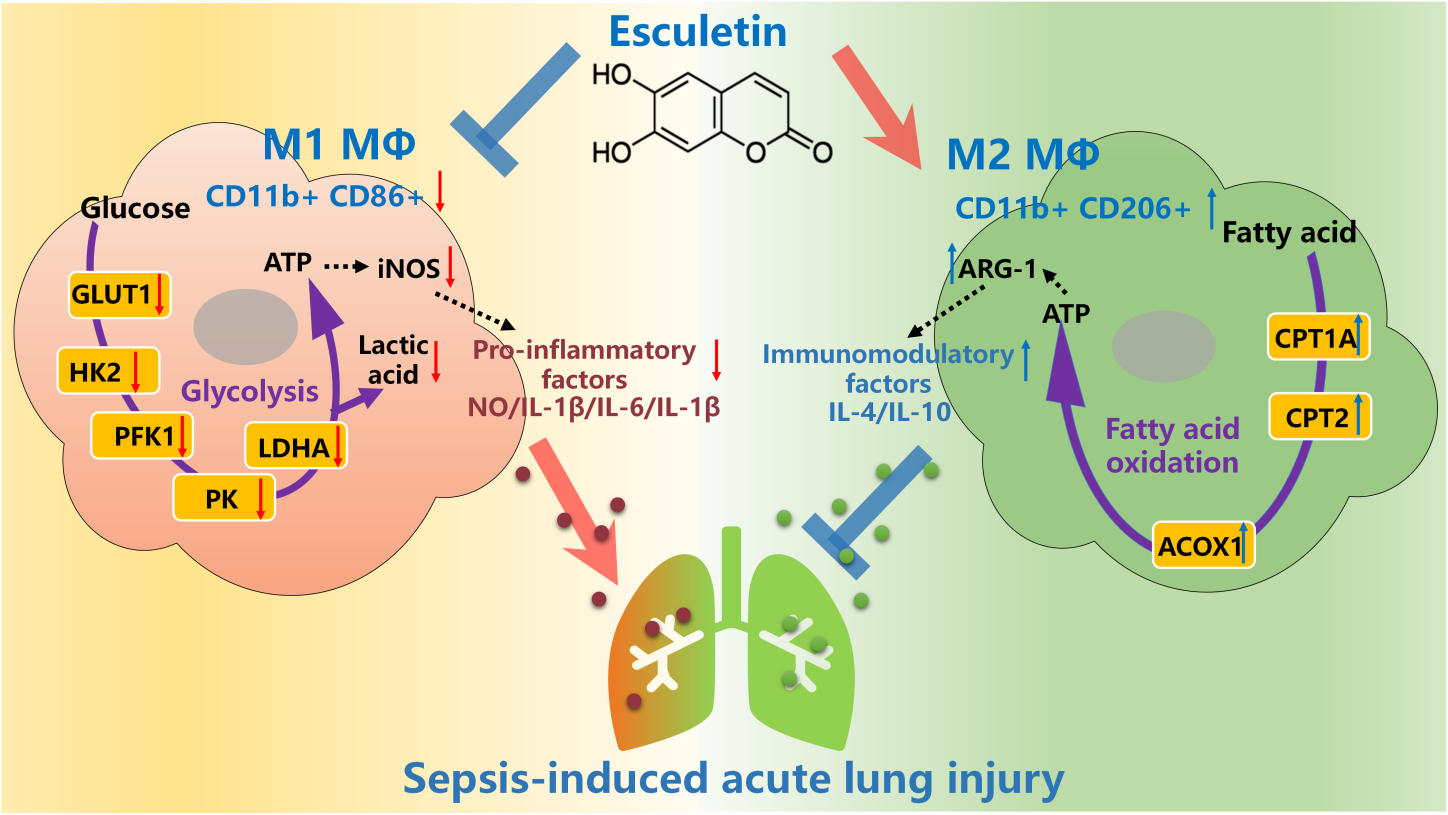
ELT can regulate macrophage polarization (inhibiting M1, while promoting M2) through metabolic reprogramming (inhibiting M1 glycolysis, while enhancing M2 fatty acid β‐oxidation), thereby improving the inflammatory response in SALI.

In summary, ELT can regulate macrophage polarization (inhibiting M1, while promoting M2) through metabolic reprogramming (inhibiting M1 glycolysis, while enhancing M2 fatty acid β‐oxidation), thereby improving the inflammatory response in SALI (Figure 9).

## AUTHOR CONTRIBUTIONS

**Feng Chen:** Investigation (equal); writing – original draft (equal). **Ning Wang:** Data curation (equal); investigation (equal); validation (equal). **Jiabao Liao:** Investigation (equal); validation (equal); visualization (equal). **Mengxue Jin:** Formal analysis (equal); investigation (equal); validation (equal). **Fei Qu:** Investigation (equal). **Chengxin Wang:** Visualization (equal). **Min Lin:** Validation (equal). **Huantian Cui:** Conceptualization (equal); writing – review and editing (equal). **Weibo Wen:** Conceptualization (equal); funding acquisition (equal); supervision (equal). **Fengjuan Chen:** Conceptualization (equal); funding acquisition (equal); writing – review and editing (equal).

## FUNDING INFORMATION

This work was supported by Jiaxing medical support fundation in critical care (7th batch, No. 2023‐1‐2), and National Natural Science Foundation of China (82060864).

## CONFLICT OF INTEREST STATEMENT

The authors declare that they have no known competing financial interests or personal relationships that could have appeared to influence the work reported in this paper.

## Supporting information


**Data S1.** Supporting information.

## Data Availability

Data will be made available on request.
