# Supplementary material for: Esculetin rebalances M1/M2 macrophage polarization to treat sepsis‐induced acute lung injury through regulating metabolic reprogramming
Source: J Cell Mol Med. 2024 Nov 13;28(21):e70178. doi: 10.1111/jcmm.70178 (PMC11558263; doi:10.1111/jcmm.70178)
Supplement: Supplementary file 1 — Data S1. Supporting information. [file JCMM-28-e70178-s001.docx]

**1 Reagents**

Esculetin (HY-N0284), 2-DG (HY-13966), etomoxir (HY-50202) was purchased from MedChemExpress LLC. Elisa kits for rat IL-1β (ml037361), IL-6 (ml102828), TNF-α (ml002859), IL-4 (IL-4), IL-10 (ml002813) were purchased from Shanghai Enzyme-linked Biotechnology Co., Ltd. Fatty acid oxidation assay kit (BR00001) were purchased from AssayGenie. NO assay kit (A013-2-1) and lactic acid assay kit (A019-2-1) were purchased from Nanjing Jiancheng Bioengineering Institute Co., LTD. Seahorse XF palmitate-BSA FAO substrate (102720-100), seahorse XF palmitate oxidation stress test kit (103693-100), and seahorse XF glycolysis stress test kit (103020-100) were purchased from Agilent Technologies, Inc. Mouse anti-CD11b (ab1211) were purchased from Abcam. Rabbit anti-CD206 (18704-1-AP), rabbit anti-CD86 (13395-1-AP), iNOS (22226-1-AP), ARG-1 (16001-1-AP) were purchased from Proteintech Group, Inc. ACTB (ab6276) and secondary antibody goat anti-rabbit IgG H&L (ab205718) were purchased from Abcam. FITC-conjugated anti-rat CD11b (FITC-65229) and rabbit anti-CD206 (18704-1-AP) antibodies for flow cytometry were purchased from Proteintech Group, Inc. PE-conjugated anti-CD86 (12-0860-83) and APC-conjugated [goat anti-rabbit IgG (H+L)](https://www.thermofisher.cn/antibody/product/Goat-anti-Rabbit-IgG-H-L-Cross-Adsorbed-Secondary-Antibody-Polyclonal/A-10931) (A-10931) antibodies for flow cytometry were purchased from Thermo Fisher Scientific Inc. Total RNA extraction, first-strand cDNA reverse transcription, polymerase chain reaction (PCR) kits and primers were obtained from TianGen Biotechnology Co., Ltd. (Beijing, China).

**2 Primer sequence used in this study**

**Table S1** Primer sequence

| Gene | Sequence (5'->3') | |
| --- | --- | --- |
| *Nos2* | Forward primer | GCCTTCAACACCAAGGTTGTC |
|  | Reverse primer | CCTGAAGGTATGCCCGAGTT |
| *Arg1* | Forward primer | AAGAAAAGGCCGATTCACCT |
|  | Reverse primer | CACCTCCTCTGCTGTCTTCC |
| *Cd206* | Forward primer | CTGCAGATGGGTGGGTTATT |
|  | Reverse primer | GGCATTGATGCTGCTGTTATG |
| *Cd86* | Forward primer | TAGGGATAACCAGGCTCTAC |
|  | Reverse primer | CGTGGGTGTCTTTTGCTGTA |
| *Glut1* | Forward primer | CCACCACACTCACCACACTC |
|  | Reverse primer | CCATAAGCACGGCAGACAC |
| *Hk2* | Forward primer | TGATCGCCTGCTTATTCACGG |
|  | Reverse primer | AACCGCCTAGAAATCTCCAGA |
| *Pfkfb1* | Forward primer | AAGCCTCTAAGAGAACAGCCTC |
|  | Reverse primer | ACTCACTGCCTCTCGTCGAT |
| *Pkm* | Forward primer | CATGCTGTCCGGAGAAACAG |
|  | Reverse primer | TCTCGAGCTATCAGGTGCTG |
| *Ldha* | Forward primer | GCTCATCGTCTCAAACCCAGTGG |
|  | Reverse primer | ACTCCCAGCCTTTCTCCCATCAG |
| *Cpt1a* | Forward primer | TCAAACCCATTCGTCTTCTGG |
|  | Reverse primer | CTGCTTATTTTTCCCTCGTGC |
| *Cpt2* | Forward primer | GCTCCGAGGCGTTTCTCA |
|  | Reverse primer | TGGCCGTTGCCAGATAGC |
| *Acox1* | Forward primer | GGGACCCATAAGCCTTTGCC |
|  | Reverse primer | CTTGTTACTACGCGGTTTCACG |
| *Actb* | Forward primer | CACCATGTACCCAGGCATTG |
|  | Reverse primer | CCTGCTTGCTGATCCACATC |
